# Parvalbumin expression and gamma oscillation occurrence increase over time in a neurodevelopmental model of NMDA receptor dysfunction

**DOI:** 10.7717/peerj.5543

**Published:** 2018-09-19

**Authors:** Ben van Lier, Andreas Hierlemann, Frédéric Knoflach

**Affiliations:** 1 Neuroscience Discovery, Pharma Research and Early Development, Roche Innovation Center Basel, F. Hoffmann-La Roche Ltd., Basel, Switzerland; 2 Department of Biosystems Science and Engineering, ETH Zürich, Basel, Switzerland

**Keywords:** Schizophrenia, Oscillation, Organotypic, Interneuron, GluN2A, Gamma, MEA, Hippocampus, NMDAR, Parvalbumin

## Abstract

Dysfunction of the *N*-methyl-d-aspartate receptor (NMDAR) is thought to play a role in the pathophysiology of neurodevelopmental diseases like schizophrenia. To study the effects of NMDAR dysfunction on synaptic transmission and network oscillations, we used hippocampal tissue of NMDAR subunit GluN2A knockout (KO) mice. Field excitatory postsynaptic potentials were recorded in acute hippocampal slices of adult animals. Synaptic transmission was impaired in GluN2A KO slices compared to wild-type (WT) slices. Further, to investigate whether NMDAR dysfunction would alter neurodevelopment in vitro, we used organotypic hippocampal slice cultures of WT and GluN2A KO mice. Immunostaining performed with cultures kept two, seven, 14, 25 days in vitro (DIV) revealed an increasing expression of parvalbumin (PV) over time. As a functional readout, oscillatory activity induced by the cholinergic agonist carbachol was recorded in cultures kept seven, 13, and 26 DIV using microelectrode arrays. Initial analysis focused on the occurrence of delta, theta, beta and gamma oscillations over genotype, DIV and hippocampal area (CA1, CA3, dentate gyrus (DG)). In a follow-up analysis, we studied the peak frequency and the peak power of each of the four oscillation bands per condition. The occurrence of gamma oscillations displayed an increase by DIV similar to the PV immunostaining. Unlike gamma occurrence, delta, theta, and beta occurrence did not change over time in culture. The peak frequency and peak power in the different bands of the oscillations were not different in slices of WT and GluN2A KO mice. However, the level of PV expression was lower in GluN2A KO compared to WT mice. Given the role of PV-containing fast-spiking basket cells in generation of oscillations and the decreased PV expression in subjects with schizophrenia, the study of gamma oscillations in organotypic hippocampal slices represents a potentially valuable tool for the characterization of novel therapeutic drugs.

## Introduction

*N*-methyl-d-aspartate receptors (NMDAR) are involved in neural plasticity and neuronal excitotoxicity. Functional NMDARs consist of two obligatory GluN1 subunits and two GluN2 or GluN3 subunits. GluN1 is present during all stages of life and in nearly all neurons whereas GluN2 plays a critical role during postnatal brain development (Monyer et al., 1994).

There are four different types of GluN2 subunits (A–D). These subunits are heterogeneously expressed in the brain and the level of expression changes during development (for review see Cull-Candy, Brickley & Farrant (2001)). In addition, NMDARs comprising different GluN2 subunits have different pharmacological and functional properties. GluN2B and GluN2D expression is already seen during embryonic stages, whereas GluN2A and GluN2C are first seen postnatal. During postnatal development, the expression of GluN2B diminishes while GluN2A rises. Both GluN2B and GluN2A are prominent in the hippocampus and cortex (Monyer et al., 1994).

Dysfunction of the NMDAR is thought to play a role in the pathophysiology of neurodevelopmental diseases, for example, schizophrenia (Cohen et al., 2015; Gonzalez-Burgos & Lewis, 2012; Weickert et al., 2013). NMDAR antagonists, including phencyclidine (PCP) and ketamine, induce schizophrenia-like symptoms in healthy subjects (Olney, Newcomer & Farber, 1999) and working memory deficits in rats (Lewis & Gonzalez-Burgos, 2006). Indeed, in subjects with schizophrenia, GluN2A is expressed at lower levels in Gamma-Aminobutyric acid (GABA) interneurons compared to normal controls (Bitanihirwe et al., 2009; Woo, Walsh & Benes, 2004).

GluN2A knockout (KO) mice display increased locomotor activity, reduced spatial learning and impaired spatial pattern processing (Kadotani et al., 1996; Kannangara et al., 2015). The increased locomotor activity of GluN2A KO mice is attenuated by antipsychotic drugs at doses that do not affect wild-type (WT) mice (Miyamoto et al., 2001). Additionally, impairment of conditioned eyeblink response was reported by Kishimoto et al. (2001). On the other hand, paired-pulse inhibition is only impaired in GluN2A KO mice when combined with a GluN2B antagonist (Spooren et al., 2004). These mice also display decreased occurrence of synaptic activity (Fu, Logan & Vicini, 2005) that could lead to impaired synaptic transmission.

Pyramidal cells in the hippocampus send NMDAR-dependent excitatory inputs to GABAergic interneurons that express the Ca^2+^-binding protein parvalbumin (PV). Fast-firing PV interneurons may be a prime target for NMDAR dysfunction. Repeated administration of NMDAR antagonists results in decreased expression of PV (Cochran et al., 2003; Kinney et al., 2006). In the prefrontal cortex, a decrease is seen in PVALB mRNA after NMDAR antagonists treatment (Cochran et al., 2003). Importantly, a decrease in the density of PV immunoreactive neurons is seen in the hippocampus, after administration of NMDAR antagonists (Keilhoff et al., 2004). PV interneurons are responsible for the generation of gamma oscillations (Cardin et al., 2009; Gonzalez-Burgos & Lewis, 2008; Sohal et al., 2009). Electroencephalography (EEG) measurements in humans show that gamma oscillations (30–100 Hz) are associated with cognitive tasks such as working memory (Howard et al., 2003). EEG recordings of schizophrenia patients show that gamma oscillations are altered compared to healthy subjects. The power in the gamma band is decreased in patients during most behavioral paradigms, although some tasks show increased power in schizophrenia (Sun et al., 2011). Besides power, synchrony of the oscillations between brain areas is also affected in schizophrenia (Uhlhaas et al., 2010). Similar to power, the synchrony can be increased and decreased in schizophrenia, depending on symptom or behavioral task. Moreover, the expression of PV is decreased in subjects with schizophrenia (Fung et al., 2010).

To study NMDAR dysfunction at the (micro) circuit level, the relatively well understood trisynaptic circuit in the hippocampus may serve as a starting point. First, two forms of synaptic plasticity have been reported after Schaffer collateral-commissural pathway tetanic stimulation. Long-term potentiation (LTP), which is thought to represent cellular correlates of learning and memory has been extensively studied in this circuit (for recent review see Nicoll (2017)). A transient, early phase of LTP decays within a period of usually 2–30 min after the initial large increase in the response size following the stimulation tetanus. This early phase is followed by sustained increase in response size that lasts until the end of the experiment (LTP). Both of these forms of synaptic plasticity are dependent on NMDAR (Bliss & Collingridge, 1993). Second, gamma oscillations can be induced in hippocampal slices with the cholinergic agonist carbachol. Carbachol exerts its effect mainly through the M1 receptor (Fisahn et al., 2002). In addition to gamma, carbachol can also induce delta, theta and beta oscillations (Arai & Natsume, 2006; Fellous & Sejnowski, 2000).

Organotypic hippocampal slice cultures can be used to study neurodevelopment (Gähwiler, 1981; Holopainen, 2005; Stoppini, Buchs & Muller, 1991). De Simoni, Griesinger & Edwards (2003) compared organotypic hippocampal slice cultures from rat at different days in vitro (DIV) to acute slices from animals at corresponding ages. They conclude that organotypic cultures at one, two and three weeks in vitro are remarkably similar to acute slices of day 14, 17 and 21, respectively. It is also important to note that α-amino-3-hydroxy-5-methyl-4-isoxazolepropionic acid (AMPA) and NMDA receptors (as well as other synaptic components) are expressed at steady-state levels after a short reduction following slicing (Bahr et al., 1995).

In the present study, we used GluN2A KO mice (Kadotani et al., 1996) to investigate NMDAR dysfunction on synaptic transmission and oscillatory activity with hypofunctioning NMDAR. We hypothesize that a KO of GluN2A leads to maturation defects in primarily PV interneurons and would alter the function of micro-circuitry in the hippocampus. We first attempted to confirm an electrophysiological phenotype of the GluN2A KO mice by performing a synaptic transmission study on acute slices. Then, immunohistochemistry was used to detect expression of PV, and microelectrode array (MEA) technology was used to record oscillatory activity of the cultures. We find that acute slices of adult GluN2A KO mice display a deficit in synaptic strength and in the early phase of LTP but not in the later phase LTP. In organotypic cultures, expression of PV increases over the first weeks in vitro, with the probability of gamma oscillations occurring increasing correspondingly. The probability of delta, theta and beta oscillations occurring does not change over time. The results show that GluN2A KO reduces PV expression, but does not alter peak frequency or peak power of delta, theta, beta or gamma oscillations.

## Materials and Methods

### Animals

Male and female WT C57 BL/6 mice were provided by Janvier Labs, Le Genest-Saint-Isle, France. GluN2A homozygous KO mice (Kadotani et al., 1996) were bred in-house after being obtained from Prof. Shigetada Nakanishi. The genotype was confirmed in a previous study (Spooren et al., 2004). All experiments were carried out under the guidelines issued under local Cantonal and Swiss federal law as approved by Kant. Veterinäramt Basel-Stadt Abteilung Tierschutz (license #196).

### Acute slices

One-year-old WT and KO mice were anaesthetized using a mixture of 2.5% isoflurane and pure oxygen and decapitated under anesthesia. The brains were rapidly removed, and hippocampi were dissected and 350 μm slices were cut with a SORVALL TC-2 tissue chopper (MTS, Liverpool, NY, USA). Slices were allowed to recover for at least 1 h at room temperature in artificial cerebrospinal fluid (aCSF) containing (in mM): 120 NaCl, 3.5 KCl, 2.5 CaCl_2_, 1.3 MgSO_4_, 1.25 NaH_2_PO_4_, 26 NaHCO_3_ and 10 d-glucose, saturated with 95% O_2_ and 5% CO_2_.

### Organotypic slices

Under sterile conditions, postnatal mice (P7) were decapitated and their hippocampi excised in ice cold dissection buffer containing (in mM): 59 Glucose, 0.086 Penicillin, 0.082 Streptomycin, 0.96 Kynurenic Acid in Hank’s Balanced Salt Solution. Slices (350 μm) of the hippocampi were cut with a McIlwain tissue chopper.

Organotypic hippocampal cultures were made on culture inserts with semipermeable membranes (Millipore PICM0RG50) according to the principles described by Stoppini, Buchs & Muller (1991). On the culture insert, four slices were placed with each one on a small cut-out of the membranes of other inserts for easier handling during experiments. Cultures were kept in an incubator with humidified atmosphere (5% CO_2_, 36 °C). Once per week, culture inserts were placed into new dishes (Corning 353801) filled with 1.2 ml culture medium (Basal Medium Eagle without l-Glutamine 48%, Hank’s Balanced Salt Solution 24%, Horse Serum 24%, Glucose 52 mM, GlutaMAX 1.29 mM, Penicillin 0.043 mM, Streptomycin 0.041 mM, B27 0.95%).

### Immunohistochemistry

This experiment involved four WT and four KO mice. From each mouse, both hippocampi were dissected at P7 and 15 slices were selected for culturing. Three cultures per mouse were taken on DIV 2, 7, 14 and 25.

The primary antibody was anti-PV (Abcam AB11427). Secondary antibody was Alexa Fluor 555 anti-rabbit (Invitrogen A31572). In addition, cell nuclei were stained with 4′,6-diamidino-2-phenylindole (DAPI) (Invitrogen D1306).

A protocol for immunostaining organotypic hippocampal cultures by Gogolla et al. (2006) was followed. First, the cultures were fixated. Cultures were placed in a solution of 4% paraformaldehyde in phosphate buffered saline (PBS) for 5 min. After that, they were placed in 20% MeOH in PBS for 5 min. After the fixation, permeabilization of the cell membranes was done by leaving the culture in 0.5% Triton X-100 for 12–18 h. To block non-specific binding of antibodies in the culture, it was left in 20% donkey serum in PBS for one day. Primary antibodies were diluted 1:1,000 in 5% donkey serum in PBS. Incubation of the culture with the primary antibody solution happened overnight by adding 500 μl of the primary antibody solution on top of the culture. The cultures were then incubated for 2 h with secondary antibody solution (diluted 1:1,000 in 5% donkey serum in PBS). Final wash was done with 5% donkey serum in PBS containing DAPI (1 μg/ml). Prolong gold antifade agent (P36934) was added to the sample before sealing the microscope slides with clear nail polish.

The stained slices were imaged with a 10× objective throughout the depth of the slice with a confocal microscope (Leica TCS SP5). The *z*-stack of images for each culture was then compressed into one image with maximum intensity projection. Further image analysis was done with Imaris (Bitplane AG, Zurich, Switzerland). Threshold intensity for signal of a voxel was set at 50 (intensity ranges 0–255). Background, therefore, was 1–49 intensity. Background was then subtracted from the signal by calculating the sum of intensity of voxels above threshold minus the average background times the number of voxels above threshold. The resulting total intensity was then log transformed.

### LTP recordings

Field excitatory postsynaptic potentials (fEPSPs) were recorded from the CA1 stratum radiatum with a glass micropipette (1–4 MΩ) containing 2M NaCl and evoked by stimulation of the Schaffer collaterals with insulated bipolar platinum/iridium electrodes. The stimulus strength was adjusted to evoke fEPSPs equal to 30% of the relative maximum amplitude without superimposed population spike. After stable baseline recordings (100 μs pulse-duration, 0.033 Hz), LTP was induced by theta burst stimulation consisting of 10 trains of five pulses at 100 Hz and intervals of 200 ms repeated twice. The duration of the stimulation pulses was doubled during the tetanus to induce maximal LTP. This stimulation protocol mimics natural firing patterns of afferent neurons in the hippocampus, optimal for NMDA-dependent LTP (Larson & Munkacsy, 2015). fEPSPs were amplified, bandpass-filtered (1 Hz–1 kHz) and stored in a computer using the pClamp software (Molecular Devices, San Jose, CA, USA). For the analysis, fEPSP slopes were expressed as a percentage of the baseline values recorded. Results from several slices were expressed as mean ± SEM.

### MEA recordings

Recordings were made on MEAs with 60 planar electrodes (Multi Channel Systems, Reutlingen, Germany). Organotypic slice cultures were placed carefully on a MEA by flipping the membrane around so that the culture was facing the electrodes. The culture was submerged in the bath with continuous 1 ml/min perfusion of aCSF (in mM: NaCl 124, KCl 2, KH_2_PO_4_ 1.25, MgSO_4_ 2, CaCl_2_ 2.5, NaHCO_3_ 26, Glucose 10) at 30 °C, saturated with 95% O_2_ and 5% CO_2_.

After a 30 s baseline, 10 μM carbachol was superfused for 1 s by using a fast application system positioned close to the slice (∼1 mm). Superfusion was controlled with an electronic open/close switch of the reservoir containing 10 μM carbachol in aCSF. Timing of 1 s was ensured by a metronome at 60 beats per minute. The response to this stimulus is then recorded over the next 30 s under continuous perfusion of aCSF. This 60 s recording constitutes one trial. The very first trial is not taken into data analysis as a control for carbachol naïve vs non-naïve reaction of the culture. This leaves five trials per culture. Trials start about 10 min after the start of the previous trial to allow the culture to return to baseline activity. The reason behind this short bolus trial design is to be able to average data within each slice. This ensures a more stable data point representing the slice in group level statistics.

### Data analysis and statistics

We used the software package MATLAB 2015a (The MathWorks Inc., Natick, MA, USA). Data management and analysis was done with an open source toolbox for electrophysiology in MATLAB called FieldTrip (Oostenveld et al., 2011) as well as custom scripts.

Raw data (20 kHz) were downsampled (including filtering) to 1,000 Hz. Then, a Butterworth high-pass 1 Hz filter was applied. Time-frequency analysis was done through short-time Fourier transform with discrete prolate spheroidal sequence (DPSS) multitaper. The frequencies analyzed were 1–500 Hz. The transform was calculated on a 1 s time window that shifts 100 ms over the entire trial (60 s). Frequencies were smoothed over ±2 Hz. All spectra were multiplied by the frequencies to correct for the 1/f noise. To make sure analysis was not affected by line hum (50 Hz) and its harmonics, ±3 Hz around these frequencies were cut-out and the gaps linearly interpolated.

In order to detect the different frequency bands where oscillations are present, we first normalized the spectra of each channel. Normalization was calculated as each value of the spectrum divided by the sum of that spectrum. Per recording, the normalized power spectra of the post-carbachol state of all 60 channels were plotted on top of each other. This way, peaks that are common among several channels stand out to the eye. The frequency range of each of these peaks was then selected by hand. Recordings were presented in random order, so the experimenter was blind to the conditions. Because not all channels are active in each frequency band, the active channels had to be separated from the non-active channels. This was done automatically by a peak detection algorithm that selected only channels that have a peak with a prominence above a threshold. We set the threshold at 0.001 normalized units based on the observation that this corresponds to a small, but distinguishable, peak in the spectrum. Peak prominence quantifies how much a peak stands out relative to other peaks. It is determined by extending a horizontal line to the left and right of a local maximum, until it crosses the signal (or the end of the signal). The minimum in both intervals is taken. Peak prominence is then how much the peak lies above the highest of the two minima.

The frequency bands were categorized as delta (1–4 Hz), theta (4–10 Hz), beta (10–30 Hz), gamma (30–100 Hz). Per frequency band of each recording, the mode of the peak frequencies amongst the active channels was taken. The band was then categorized according to within which range this peak frequency fell.

Because pictures were made of each culture on the MEA, it is possible to see which electrodes are recording in which area of the hippocampus. We categorized channels as being either on DG, CA3, CA1.

We performed binomial (present/absent) logistic regression to model the probability of delta, theta, beta and gamma occurrence (dependent variable) over the different independent variables (i.e., DIV, genotype and area). The full model includes all independent variables, and the interactions between them, as potential effects/predictors on the dependent variable. Other models consist of fewer effects (e.g., only DIV and genotype, but not their interaction). The “null model” includes no independent variables. The Akaike Information Criterion (AIC) gives an indication of goodness-of-fit of the regression and includes a penalty for models with more independent variables to avoid overfitting. For each oscillation range, the model with the lowest AIC is chosen for further assessment of the statistical significance of the effects included in that model.

Other statistical analyses were done with ANOVA. The first step was to check whether the dependent variable is affected differently depending on particular combinations of independent variables. Such an interaction between the independent variables makes it impossible to conclude about a general effect of each independent variable alone. When an interaction is found to be statistically significant, the simple effect of one independent variable is investigated per level of the other independent variables.

## Results

### Impaired synaptic transmission in acute slices of KO mice

**Figure 1:**
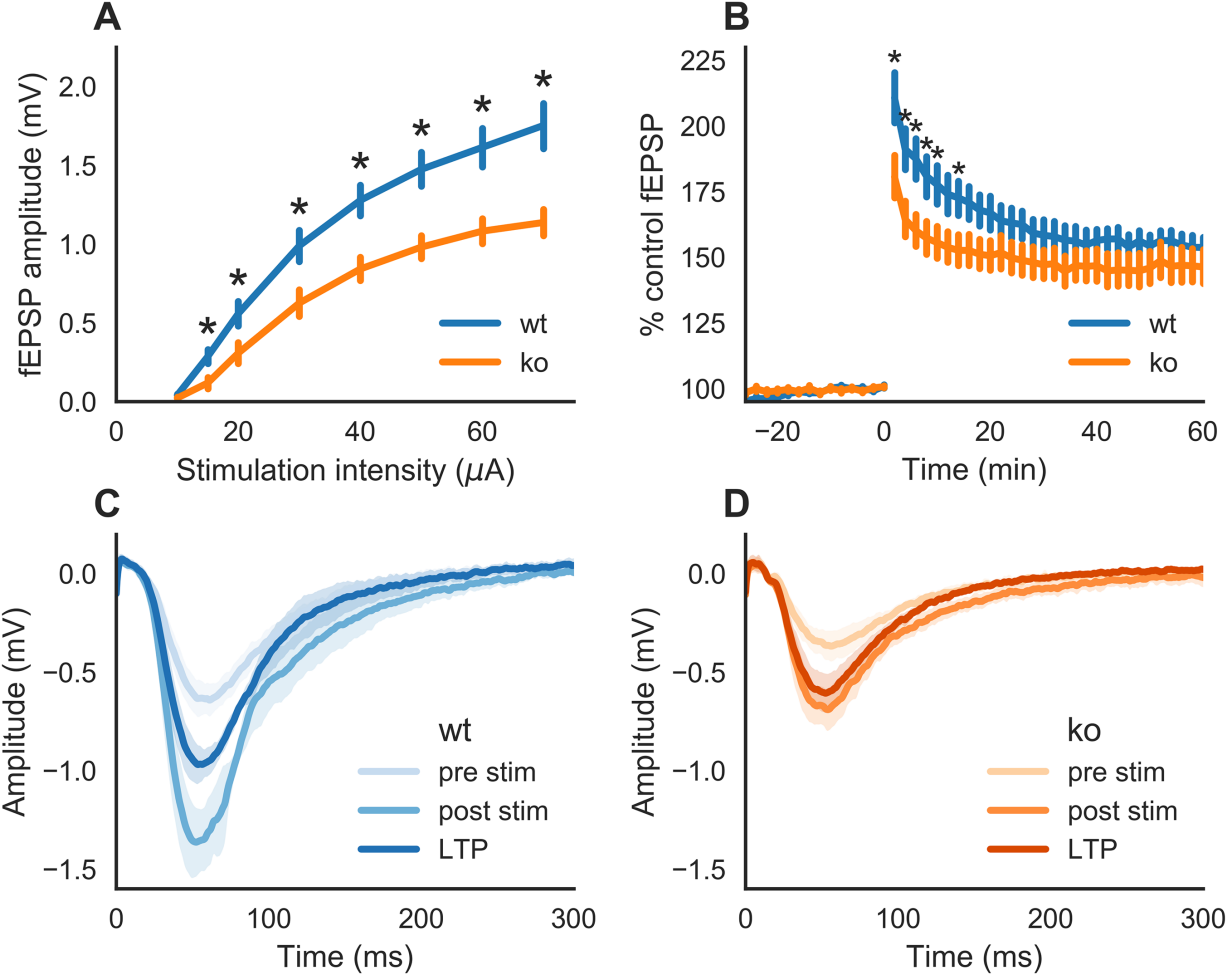
Synaptic transmission in hippocampal slices of wild-type (WT) and GluN2A homozygous knockout (KO) mice. (A) Input-output curve. Schaffer collaterals were stimulated every 30 s with increasing intensities. The slices of KO mice showed a markedly reduced response in CA1 as compared to those of WT mice. (B) Time course of field excitatory postsynaptic potentials (fEPSPs) recorded in the CA1 after Schaffer collaterals stimulation (30 s intervals) of the same intensity. A theta burst stimulation was issued to the Schaffer collaterals at time 0. A smaller increase in fEPSPs after the stimulation was observed in slices of KO as compared to WT mice. (C) Mean (±SEM) fEPSP recorded from WT slices during baseline (pre-stim), 2 min (post-stim) and 1 h after theta burst stimulation. (D) Mean (±SEM) fEPSP recorded from KO slices during baseline (pre-stim), 2 min (post-stim) and 1 h after theta burst stimulation. The recordings were performed with hippocampal slices from three adult animals (eight slices of WT and 11 slices of KO mice). Error bars indicate SEM. Statistical significance marked * is *p* < 0.05.

We first examined whether slices of KO mice would exhibit an impairment in synaptic transmission as reported earlier (Kannangara et al., 2015; Kiyama et al., 1998; Sakimura et al., 1995). fEPSPs were generated by stimulation of the Schaffer collaterals to CA1 pathway. A two-way ANOVA indicated a statistically significant interaction between genotype and stimulation intensity on the stimulation response (*F*(1,148) = 16.202, *p* < 0.0001). This means that (the strength of) the effect of genotype is not the same on all levels of stimulation intensity. As represented in Fig. 1A, fEPSPs in slices from KO mice had smaller response amplitudes than WT at all stimulation intensities tested, except at the lowest stimulation intensity (10 μA, *p* = 0.0810). At a stimulation intensity of 15 μA, the fEPSP amplitude in slices of KO (0.11 mV ± 0.03, *n* = 11) was lower than that of WT (0.28 mV ± 0.04, *n* = 8) with *p* = 0.0116. At the highest stimulation intensity tested, 70 μA, the fEPSP amplitudes were 1.13 mV ± 0.08 (*n* = 11) and 1.75 mV ± 0.14 (*n* = 8) in slices from KO- and WT-mice, respectively (*p* = 0.0013).

Therefore, slices of KO mice displayed a deficit in synaptic strength compared to those of WT mice.

Next, we tested whether LTP can be induced in slices of KO mice. fEPSPs were potentiated after stimulation of the Schaffer collaterals with a theta burst stimulation paradigm (Fig. 1B). Deficits in LTP were observed in slices of KO mice (one-way ANOVA on genotype: *F*(1,568) = 60.521, *p* < 0.0001). Post hoc testing revealed that the difference between the genotypes is only during the early phase of LTP. Statistically significant lower potentiation for KO was seen on time 2, 4, 6, 8, 10 and 14 min after tetanus stimulation (respectively, *p* = 0.0432, *p* = 0.0261, *p* = 0.0145, *p* = 0.0339, *p* = 0.0390 and *p* = 0.0446). The average potentiation during the 2–14 min post-tetanus interval in KO was 160.0% ± 9.6 (*n* = 11) and for WT it is 184.0% ± 13.2 (*n* = 8). However, at 1 h after the tetanic stimulation (LTP), there were no significant differences (*p* = 0.409) in the potentiation of fEPSP slopes in KO slices (164% ± 5.2, *n* = 11) and WT (160% ± 9.6, *n* = 8). The average (±SEM) of all traces of fEPSPs from the baseline, 2 min post-stim and LTP (1 h) are shown in Fig. 1C for WT slices and in Fig. 1D for KO slices.

Therefore, GluN2A subunit is required for a normal synaptic transmission and plasticity.

### PV expression increases over time and is reduced in KO cultures

**Figure 2:**
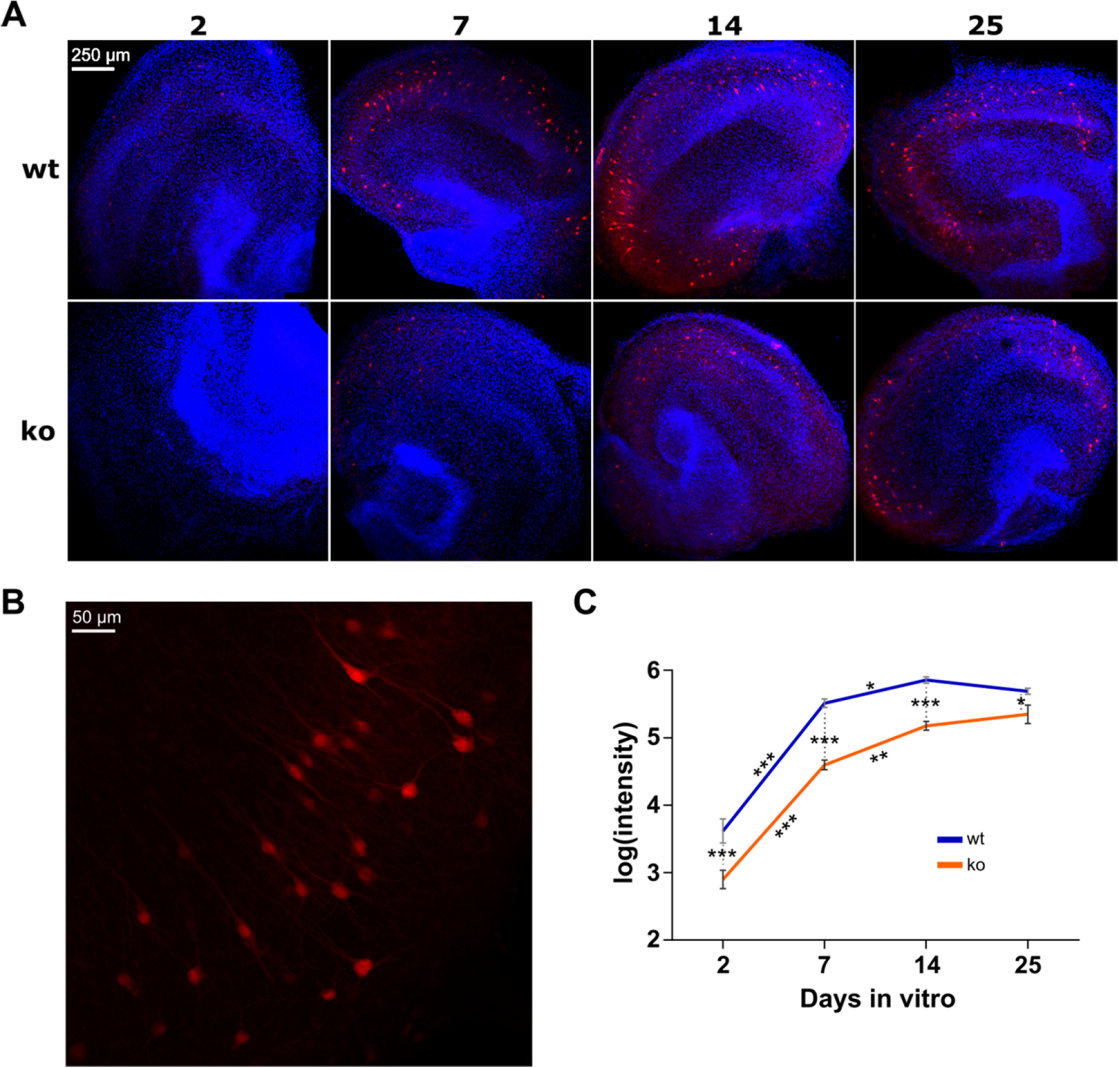
Immunostaining of parvalbumin in organotypic hippocampal cultures of GluN2A homozygous knockout (KO) and wild-type (WT) mice. (A) Representative examples of analyzed images for each condition (genotype in rows, days in vitro in columns). Red is immunoreactivity of parvalbumin antibody (Alexa Fluor 555). Blue is cell nuclei stained with DAPI. (B) Close-up example of a WT culture on DIV 14. (C) Quantification of parvalbumin expression. Two-way ANOVA on the log transformed signal intensity for genotype and DIV showed a significant interaction. Post hoc testing resulted in statistically significant reduction of signal in KO on each DIV. One-way ANOVA was conducted per genotype to quantify the effect of DIV in subsequent pairs (i.e., 2–7, 7–14 and 14–25). For both WT and KO, signal intensity is statistically significantly increased from DIV 2 to 7 and from 7 to 14, but not from 14 to 25. Mean (±SEM) shown. Statistical significance marked ***p < 0.0001, **p < 0.001 and *p < 0.05.

In vivo, the expression of PV starts postnatal and increases until adolescence and is reduced in subjects with schizophrenia (Fung et al., 2010). We performed immunohistochemistry to investigate the expression of PV in organotypic hippocampal cultures of WT and KO mice on 2, 7, 14 and 25 DIV (Fig. 2). On each DIV, 12 WT cultures and 12 KO cultures were used.

Two-way ANOVA was used to examine the effect of DIV and genotype on the expression of PV. There was a statistically significant interaction between DIV and genotype on the intensity of PV fluorescence (*F*(3, 86) = 2.894, *p* = 0.0399). This means that the strength of the effect of genotype is not equal on the individual DIV (or vice versa). Therefore, we investigated the simple effects of genotype per individual DIV and vice versa.

To assess the effect of genotype on each DIV, a post hoc test with Holm-Sidak correction was conducted. A statistically significant decrease of PV expression in KO was found on all DIV. On DIV2 *p* < 0.0001, on DIV7 *p* < 0.0001, on DIV14 *p* < 0.0001 and on DIV25 *p* = 0.0163.

To assess whether the expression of PV changes over DIV within WT and KO mice, one-way ANOVA was performed for each genotype. For both WT and KO the test showed statistically significant differences in intensity between the DIV (respectively, *F*(3, 46) = 111.1, *p* < 0.0001 and *F*(3, 40) = 99.97, *p* < 0.0001). Post hoc testing with Holm-Sidak correction was done to determine differences between subsequent DIV pairs (i.e., 2–7, 7–14 and 14–25). In WT, there was a statistically significant increase from DIV2 to DIV7 (*p* < 0.0001) as well as from DIV7 to DIV14 (*p* = 0.0330). DIV14–DIV25 did not change (*p* = 0.2318). The same changes were observed in KO: both the DIV2–DIV7 and DIV7–DIV14 pairs increased with time (respectively, *p* < 0.0001 and *p* = 0.0006) whereas DIV14–DIV25 did not change (*p* = 0.2390).

Therefore, PV expression increases during the first two weeks in organotypic cultures, and is reduced overall in cultures of KO mice.

### Gamma oscillation occurrence increases over time

**Figure 3:**
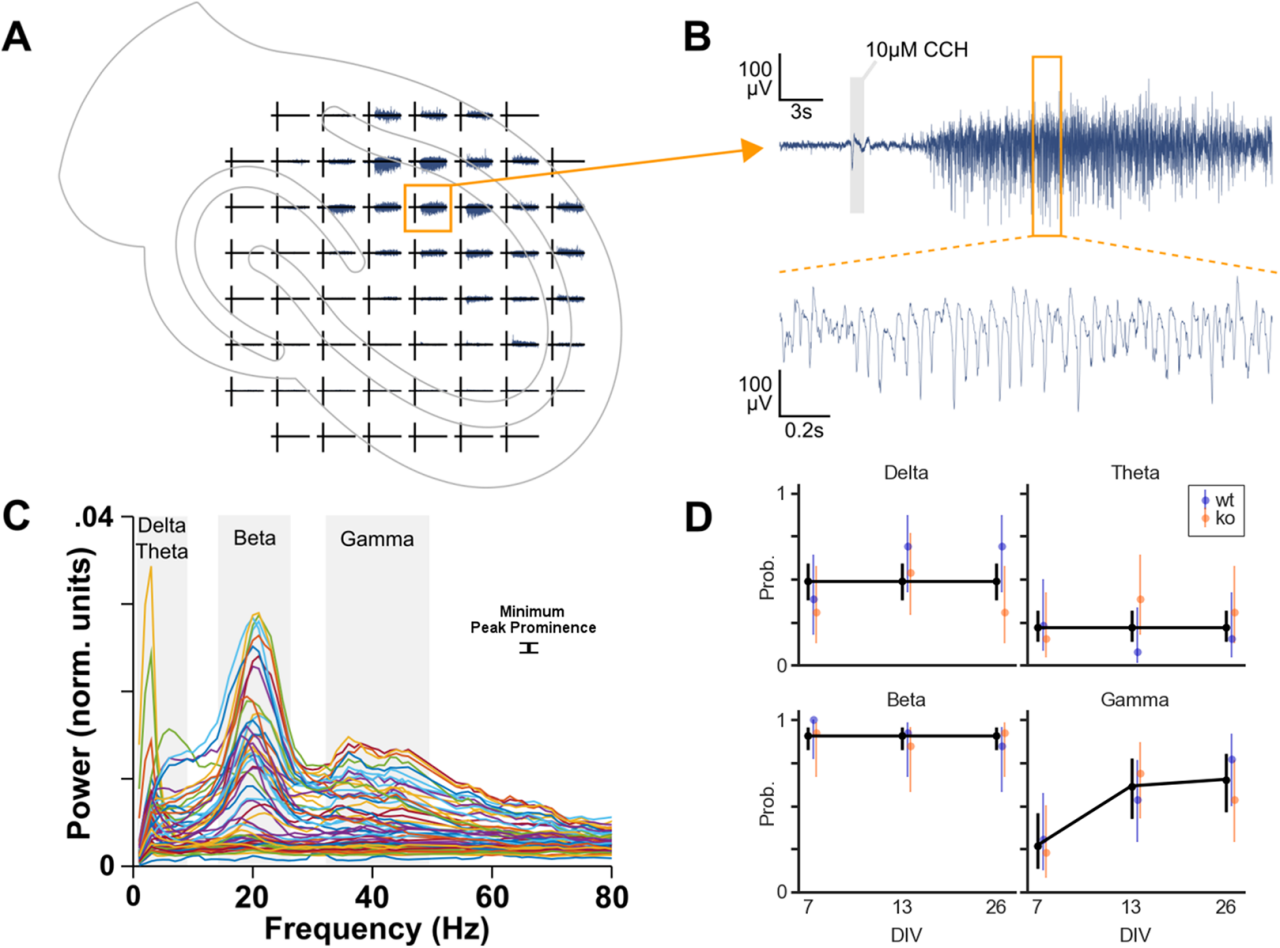
Oscillation analysis of organotypic hippocampal cultures on microelectrode arrays. (A) Example recording of a WT culture on a 60 channel MEA with the approximate location of the culture. In this case, the locus of activity is in the CA1 region. (B) One channel from plot A. A small artifact is seen at the 1 s application of 10 μM carbachol, but this is not part of analyzed data. Carbachol clearly induces strong oscillatory activity. (C) Normalized power spectral densities of 60 channels. The frequency bands of peaks in the spectra were manually selected. Within each band, a minimum peak prominence of 0.001 normalized units was the threshold to determine active channels. The mode of the peak frequencies of active channels within a band was used to categorize the band as delta (1–4 Hz), theta (4–10 Hz), beta (10–30 Hz) or gamma (30–100 Hz). (D) Estimated probability of the occurrence of each oscillation over days in vitro. Binomial logistic regression was performed to investigate the effect of genotype and DIV on the occurrence of each oscillation type. DIV predicted the occurrence of gamma oscillations but not the other oscillations. Genotype had no effect on oscillation occurrence.

In total 78 cultures were recorded on MEAs: 39 cultures from four WT mice and 39 cultures from six KO mice. Cultures were also recorded at different DIV. Per genotype, 13 cultures were recorded on DIV 7, 13 and 26. To investigate whether each of the four oscillation bands is more likely to occur due to genotype, DIV or area we performed logistic regression (Fig. 3).

Initial analyses were done with a model including genotype, DIV, area and their interactions as predictors. For theta and beta, these full models were not statistically significantly different from the null model (*p* = 0.0929 and *p* = 0.8336, respectively). However, delta and gamma models did differ from their null models (*p* = 0.0256 and *p* < 0.0001, respectively).

Because none of these models showed area to be a useful predictor, we decided to compress the three observations for DG, CA3 and CA1 per culture into a single observation per culture. We removed the area predictor from the data by categorizing a culture as having an oscillation occurring if at least one of the areas was active in that specific band.

Subsequently, the logistic regression analyses were run with genotype, DIV and their interaction as predictors. No effect of the predictors was found for delta, theta and beta oscillations. Overall, delta oscillations occurred in 49% of the cultures, theta occurred with a probability of 22% and beta occurred in almost all the cultures (91%). The occurrence of gamma oscillations, on the other hand, was found to be affected by DIV (*p* = 0.024), see Fig. 3D. Post hoc analysis revealed that the probability of gamma occurrence increased statistically significantly between DIV 7 (27%) and DIV 13 (62%), but not between DIV 13 and DIV 26 (65%).

Therefore, the occurrence of gamma oscillations in organotypic cultures (of WT and KO) after application of carbachol increases during the first two weeks in vitro.

### No changes in peak frequency and peak power

**Figure 4:**
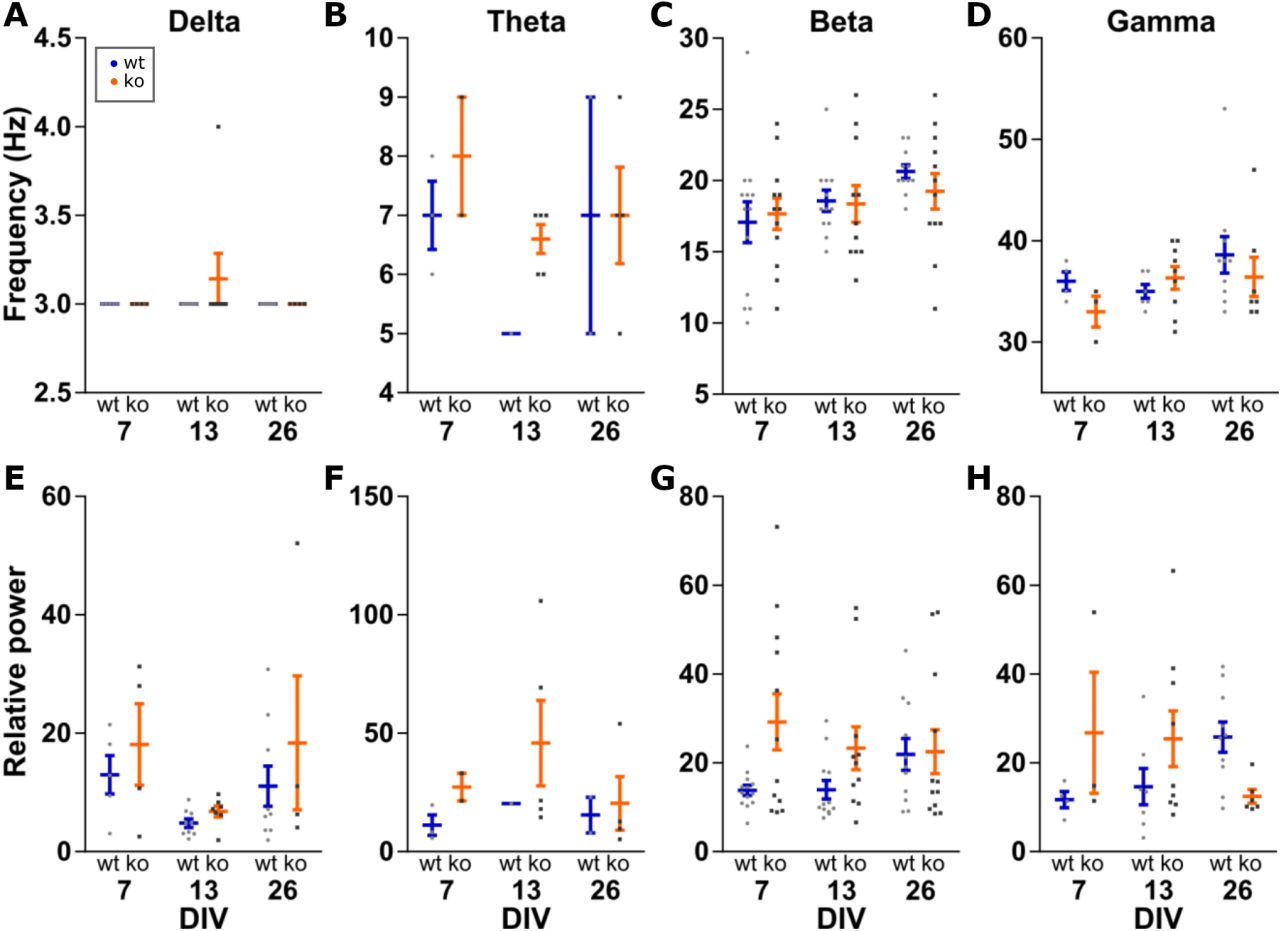
Analysis of peak frequency and peak power. Peak power is made relative to the mean power at 200–250 Hz to control for changes in power along the entire spectrum. Two-way ANOVA for genotype and DIV shows no statistically significant differences in peak frequency (A–D) or peak power (E–H) for any of the four oscillations bands. All data points shown with mean ± SEM.

Further analysis was done on the peak of the detected oscillations. Within an oscillation band, the peak frequency was compared between the conditions. Peak power was made relative to the mean power between 200 and 250 Hz to control for changes in power along the entire spectrum. This relative peak power was then compared between conditions (Fig. 4). Peak frequency and peak power are each a family of four tests, therefore we set α = 0.0125 per test.

Delta peak frequency was stable at 3 Hz among all conditions. For delta peak power, no effects of genotype (*F*(1, 32) = 1.934, *p* = 0.1739) or DIV (*F*(2, 32) = 3.799, *p* = 0.0331) were detected.

No effects of genotype (*F*(1, 11) = 1.260, *p* = 0.2855) or DIV (*F*(2, 11) = 1.507, *p* = 0.2639) were seen for theta peak frequency, as well as peak power (genotype: *F*(1, 11) = 1.034, *p* = 0.3311 and DIV: *F*(2, 11) = 0.3517, *p* = 0.7111).

No effect for beta peak frequencies was detected either (genotype: *F*(1, 65) = 0.1364, *p* = 0.7131 and DIV: *F*(2, 65) = 2.664, *p* = 0.0773). Similarly, no effect of genotype (*F*(1, 65) = 6.249, *p* = 0.0150) and DIV (*F*(2, 65) = 0.4111, *p* = 0.6646) for beta peak power.

For gamma oscillations, the peak frequency was not changed between the conditions (genotype: *F*(1, 33) = 0.7697, *p* = 0.3867 and DIV: *F*(2, 33) = 1.506, *p* = 0.2366). For gamma peak power, neither genotype (*F*(1, 33) = 0.8005, *p* = 0.3774) nor DIV (*F*(2, 33) = 0.0202, *p* = 0.9801) showed a statistically significant effect.

Therefore, the frequency and power of carbachol induced oscillations in the delta, theta, beta and gamma band do not change in KO or over time in our organotypic cultures.

## Discussion

In an effort to analyze a potential in vitro neurodevelopmental model of NMDAR dysfunction, we applied organotypic culturing, extracellular LTP recording, immunohistochemistry and MEA technology.

### Impaired early phase LTP in acute slices of KO mice

First, we examined whether a physiological phenotype can be found in KO mice. For this, we used acute hippocampal slices of WT and KO mice. We have found that the synaptic strength at CA3-CA1 synapses is reduced in slices of KO mice. This also resulted in smaller early phase of LTP in slices of KO mice. These data confirm the impairment in synaptic transmission observed in previous reports (Kannangara et al., 2015; Kiyama et al., 1998; Sakimura et al., 1995). However, the theta burst stimulation used in the present study did not reveal a statistically significant difference in later phase LTP in slices of KO vs WT mice. In the present study, we have used a theta-burst stimulus to induce LTP, as compared to stronger stimulation paradigms used in the other reports. An even stronger tetanic stimulation was shown to restore the impairment in LTP seen in slices of KO mice (Kiyama et al., 1998). Alternatively, the stronger impairment of LTP might be due to the presence of picrotoxin in the bathing solution that blocks GABA_A_ mediated transmission (Kannangara et al., 2015; Sakimura et al., 1995). The physiologically relevant, theta-burst stimulus used in the present study is compatible with neural oscillation patterns seen in the EEG during memory formation and triggers activation of NMDA receptors to induce LTP (Larson & Munkacsy, 2015). The impairment in the early phase LTP due to reduced activity and/or expression of GluN2A receptors may account for the abnormalities of oscillatory activity observed in neurodevelopmental disorders like schizophrenia (Sun et al., 2011).

### PV expression increases over time and is reduced in KO cultures

We used organotypic hippocampal slice cultures of WT and KO mice to investigate neurodevelopment in vitro. The cultures were made from P7 mice and the PV expression measurements were performed on DIV 2, 7, 14 and 25. So, a straightforward comparison to in vivo time-courses would be DIV + 7 days.

In mice, neocortical PV immunoreactivity is first detected at P10 (Del Rio et al., 1994). This maturation was not completed until adult stages. In the rat hippocampus, PV mRNA is not detected during the first postnatal week. PV expression then ramps up over the next two weeks to reach adult levels (Alcantara, Ferrer & Soriano, 1993; De Lecea, Del Rio & Soriano, 1995; Seto-Ohshima et al., 1990). This time course of PV mRNA expression fits with the present data on organotypic cultures with PV protein expression. At DIV 2 (+P7) very low PV expression is found which then increases until DIV 14 (+P7) in both WT and KO. In humans, PV expression also takes place at a later stage. Starting postnatal, PV expression increases sharply over the first two years and then stays level throughout life (Fung et al., 2010).

While showing a similar pattern of PV expression to WT, the expression was reduced in KO on all DIV. This finding is interesting in light of the NMDAR hypofunction theory of schizophrenia. A consistent finding in post-mortem tissue of subjects with schizophrenia is a reduced amount of PV (Fung et al., 2010; Hashimoto et al., 2008; Mellios et al., 2009). NMDAR antagonism by repeated administration of ketamine or PCP reduces the number of PV positive interneurons in rat hippocampus (Abdul-Monim, Neill & Reynolds, 2007; Keilhoff et al., 2004). The PV reduction due to ketamine is seen in primary cultures as well (Behrens et al., 2007; Kinney et al., 2006). Interestingly, this effect was reproduced by GluN2A antagonist NVP-AAM077 (Kinney et al., 2006).

Belforte et al. (2009) showed that a postnatal, but not adult, ablation of GluN1 in interneurons reduced PV expression and induced behavioral impairments related to schizophrenia. These impairments include psychomotor agitation, reduced preference for sweet solution as well as deficits in spatial working memory and short-term social memory. These observations raise the importance of the disturbed development triggered by NMDAR hypofunction. Our data do not allow conclusions on late KO of GluN2A. However, the immunohistochemistry data does suggest disturbed development of interneurons due to GluN2A KO.

### Oscillation occurrence is modified over time

#### Gamma

In addition to PV expression, gamma oscillations also show a late, protracted development during childhood and adolescence in humans (Uhlhaas et al., 2010). Similarly, gamma oscillations appear after about one week postnatal in rodents. Consequently, they develop over the course of the first three postnatal weeks (Minlebaev et al., 2015), which corresponds to adolescence. This matches the profile of gamma oscillation occurrence observed with organotypic cultures in the present study.

It is thought that there are two gamma generators in the hippocampus. One is in DG, which depends on input from entorhinal cortex. The other is in CA3, which then projects to CA1 (Csicsvari et al., 1999). The entorhinal cortex was included in the cultures used here. Therefore, a contribution of DG in the recorded oscillations cannot be ruled out. Our data show occurrence of gamma oscillations at a similar level in every region examined.

#### Delta

We also looked at other frequency ranges including delta, theta and beta ranges. Delta oscillations are observed in vivo in rodent cortex and hippocampus and are thought to be generated by the interaction of pyramidal cells and interneurons (Fellous & Sejnowski, 2000). These authors found delta oscillations induced by carbachol in acute rat hippocampal slices. By isolating the CA3, CA1 and DG areas, they also conclude that the origin of the delta activity is in CA3. In contrast, our data show no difference in the likelihood of occurrence of delta in these regions after application of carbachol. We also do not find a difference in the occurrence of delta over DIV 7, 13 and 26, with roughly half of the cultures showing delta overall.

#### Theta

In vivo work suggests a major role of cholinergic inputs from the septum in generating hippocampal theta oscillations (Brazhnik & Fox, 1997). However, in vitro work shows that hippocampal pyramidal cells exhibit sub threshold membrane potential fluctuations at theta frequencies (Leung & Yim, 1991). Indeed, acute slices of hippocampus without septal input show theta activity when cholinergic receptors are activated (Konopacki et al., 1987). They seem to be generated in CA3 and then propagate to CA1 and DG (Williams & Kauer, 1997). This may explain why we did not find a particular area displaying theta oscillations more than another area. Fischer et al. (2002) investigated theta and gamma oscillations induced by cholinergic agonists in organotypic hippocampal cultures, confirming the ability of hippocampus to oscillate intrinsically (Fischer, 2004). These studies were done with cultures three to six weeks old, thus not allowing a direct comparison with our data which show that theta occurrence probability is not changed over DIV 7, 13 and 26.

#### Beta

In almost all of the organotypic cultures recorded, we detected beta frequency activity. Kopell et al. (2000) suggest that beta oscillations display properties that make them more useful for long range synchronization between brain areas, whereas gamma would be more useful in relatively local computation. The oscillations in the beta range seen in our data are around 20 Hz. One interpretation could be that these are in fact slow gamma oscillations. Higher temperatures of the recording milieu are directly related to higher frequencies (Schneider et al., 2015). Because our recording temperature was 30 °C, we are confident that beta frequencies seen in the cultures is not simply slow gamma. More so because actual gamma frequencies occur often in the data as well. In another study by Shimono et al. (2000), beta (∼20 Hz) is induced by carbachol in rat hippocampal slices as recorded on MEAs. Carbachol-induced beta oscillations in rat hippocampal slices are generated in CA3 and spread to CA1 and DG (Arai & Natsume, 2006). This fits with the current result that the area has no effect on the occurrence of beta oscillations in the various regions examined.

#### Genotypes

The regression modeling did not predict a difference between WT and KO in the probability of oscillation occurrence for any of the four bands. The genotypes were both expected to show oscillations. EEGs from subjects with schizophrenia display gamma oscillations; however, they are irregular compared to healthy subjects. The abnormalities may occur in power and synchrony of the gamma oscillations, and both can be increased or decreased. This depends on the cognitive task during which the EEG is recorded (Lee et al., 2003; Uhlhaas et al., 2010).

### No changes in peak frequency and peak power

We explored whether the peak frequency and peak power relative to power at 200–250 Hz changes within the oscillation bands. PV containing interneurons are important in gamma oscillations (Cardin et al., 2009; Sohal et al., 2009). PV is reduced, and gamma oscillations are altered, in subjects with schizophrenia and due to NMDAR antagonism (Cohen et al., 2015). Therefore, we expected to see altered gamma oscillations in our KO mice showing PV reduction. However, for both frequency and power, we report no statistically significant changes over DIV and genotype for all of the four oscillation bands.

Carlén et al. (2012) studied GluN1 KO in PV cells only and found an increase in spontaneous gamma (36–44 Hz) power in vivo. Because GluN1 subunits are a part of all NMDARs, this KO may have stronger consequences for the PV neurons than our (global) GluN2A KO. However, other researchers have also found no change in frequency and power of gamma oscillations induced by kainate or carbachol due to NMDAR antagonism (ketamine) in hippocampus in vitro (Cunningham et al., 2006; Dickinson et al., 2003; Roopun et al., 2008). Besides power, alterations in gamma synchronization are also found in schizophrenia patients (Lee et al., 2003; Uhlhaas et al., 2010). In order to investigate this, however, hippocampal slices are not suitable as the synchronization in question relates to connectivity over larger distances (i.e., brain areas).

*N*-Methyl-d-Aspartate receptor antagonism and schizophrenia are also known to affect glutamate decarboxylase 67 (GAD67) and GABA transporter 1 (GAT1) (Kinney et al., 2006; Volk et al., 2000, 2001). GAD67 synthesizes GABA from glutamate and GAT1 is a transporter for reuptake of GABA from the synaptic cleft. These findings indicate an impairment of GABAergic transmission. Such an impairment may reduce gamma power because of the important role of PV containing GABA interneurons in generating gamma oscillations. Because the function of PV is that of a calcium buffer (Plogmann & Celio, 1993; Schwaller, 2007), an intriguing thought is that the reduction seen in PV expression is a compensatory mechanism (Lewis, Hashimoto & Volk, 2005). Calcium plays an important role in neurotransmitter release. Less PV may leave more calcium unbound which leads to enhanced release of GABA from the interneurons. Indeed, gamma oscillation power is increased in PV KO mice (Vreugdenhil et al., 2003). Thus, the lack of gamma oscillation alterations, despite the PV reduction in our KO, is perhaps a sign of successful compensatory activity.

## Conclusion

To test the validity of KO mice as a model for NMDAR hypofunction, we performed field potential and oscillation recording experiments in acute and cultured hippocampal slices, respectively. Compared to WT, acute slices of adult KO mice show a deficit in synaptic transmission. We next sought to find evidence for neurodevelopmental effects of GluN2A KO using organotypic cultures as a model.

In organotypic hippocampal cultures, PV expression and gamma oscillation occurrence increase strongly over the first two weeks in vitro. The expression and occurrence effects subsequently plateau over the third and fourth week in vitro. These observations may be analogous to in vivo maturation. Additionally, the cultures of KO mice showed a similar curve of PV expression but at a lower level compared to WT.

Thus, we conclude that GluN2A KO mice show an interesting phenotype and that organotypic hippocampal cultures are valuable in studying neurodevelopment in the context of schizophrenia.

## Additional Information and Declarations

### Data Availability

The following information was supplied regarding data availability: van Lier, Ben (2018): MEA. figshare. Fileset. DOI 10.6084/m9.figshare.6882788.v1; van Lier, Ben (2018): IHC. figshare. Fileset. DOI 10.6084/m9.figshare.6941468.v1; van Lier, Ben (2018): LTP. figshare. Dataset. DOI 10.6084/m9.figshare.6941474.v1.
